# Biomaterials Based on Bee Products and Their Effectiveness in Soft Tissue Regeneration

**DOI:** 10.3390/ma18122689

**Published:** 2025-06-07

**Authors:** Corina Dana Dumitru, Ionela Andreea Neacșu, Ovidiu Cristian Oprea, Ludmila Motelica, Bianca Voicu Balasea, Cornelia-Ioana Ilie, Florica Marinescu, Alexandra Ripszky, Silviu-Mirel Pituru, Ecaterina Andronescu

**Affiliations:** 1 Department of Science and Engineering of Oxide Materials and Nanomaterials, Faculty of Chemical Engineering and Biotechnology, National University of Science and Technology Politehnica Bucharest, 1-7 Gheorghe Polizu, 011061 Bucharest, Romania; corina.dumitru95@yahoo.com (C.D.D.); cornelia_ioana.ilie@upb.ro (C.-I.I.);; 2 National Research Center for Micro and Nanomaterials, National University of Science and Technology Politehnica Bucharest, 313 Splaiul Independentei, 060042 Bucharest, Romania; ludmila.motelica@upb.ro; 3 Department of Inorganic Chemistry, Physical Chemistry and Electrochemistry, Faculty of Chemical Engineering and Biotechnologies, National University of Science and Technology Politehnica Bucharest, 1-7 Gheorghe Polizu, 011061 Bucharest, Romania; 4 Academy of Romanian Scientists, 3 Ilfov Street, 050044 Bucharest, Romania; 5 Research Center for Advanced Materials, Products and Processes, National University of Science and Technology Politehnica Bucharest, 313 Splaiul Independentei, 060042 Bucharest, Romania; 6 Interdisciplinary Center for Dental Research and Development, “Carol Davila” University of Medicine and Pharmacy, 6 Traian Vuia Str., 020956 Bucharest, Romania; bianca.voicu-balasea@drd.umfcd.ro (B.V.B.); alexandra.ripszky@umfcd.ro (A.R.);; 7 Department of Botany and Microbiology, Faculty of Biology, University of Bucharest, 1-3 Aleea Portocalelor Str., 060101 Bucharest, Romania; florica.marinescu@bio.unibuc.ro; 8 The Research Institute of the University of Bucharest, University of Bucharest, 90-92 Panduri, 050663 Bucharest, Romania; 9 Department of Biochemistry, Faculty of Dental Medicine, “Carol Davila” University of Medicine and Pharmacy, 17-23 Plevnei Str., 020021 Bucharest, Romania; 10 Department of Organization, Professional Legislation and Management of the Dental Office, Faculty of Dental Medicine, “Carol Davila” University of Medicine and Pharmacy, 17-23 Plevnei Str., 020021 Bucharest, Romania

**Keywords:** alginate, chitosan, natural/bee products, biomaterials, wound healing

## Abstract

The increasing prevalence of antibiotic-resistant bacteria has stimulated the search for alternative antimicrobial agents with greater efficacy, low toxicity, and minimal resistance potential. Natural products, such as honey, propolis, and royal jelly, have shown promise due to their biological properties. The integration of natural products like honey and propolis in biomaterials represents a synergistic approach to combat the growing threat of resistant bacterial infections while improving wound care and soft tissue engineering applications. In the present work, we obtained sodium alginate films based on honey, propolis, royal jelly, and their mixture coated with chitosan for soft tissue regeneration. SEM showed that adding bee products altered surface morphology, affecting roughness, porosity, and microstructure. Spectral analysis confirmed specific chemical bonds, while thermal studies indicated a good stability up to 115 °C. The antimicrobial activity was evaluated against Gram-positive (*Enterococcus faecalis*, *Staphylococcus aureus*), Gram-negative (*Escherichia coli*, *Pseudomonas aeruginosa*) and yeast strains (*Candida albicans*), with growth inhibition zone diameters up to 12 mm. In vitro cytotoxicity studies, made on human gingival fibroblasts, suggested good biocompatibility. Antimicrobial assays showed that films containing propolis tincture, alone or as a mixture, were most effective against pathogens. Future research will focus on formulation optimization for biomedical use.

## 1. Introduction

The skin is the human body’s largest organ, acting as a protective barrier against environmental factors. Skin damage causes disruption of epithelial integrity and loss of physiological functions. Wound healing is a complex and dynamic process that restores tissue integrity and function [1]. An ideal wound dressing should meet several key criteria: it must absorb wound exudates and maintain optimal moisture at the wound site, prevent bacterial infections, and demonstrate biocompatibility without causing allergies or toxicity, leading to the completion of the healing process [2,3]. The dressing should also offer antibacterial properties and a soft texture to enhance comfort and effectiveness [4].

Wound dressings based on biomaterials have excellent properties, such as antibacterial activity, moisture retention, hemostasis promotion, and anti-inflammatory effects. Incorporating natural antibacterial agents into these wound dressings can further enhance their properties and promote faster wound healing [5,6,7].

Chitosan, commonly extracted from crustaceans’ shells, exhibits hemostatic, antibacterial, and tissue-regenerative properties. Chitosan-based hydrogels are emerging as innovative wound dressings, suitable even for complex tissue damage [8].

Sodium alginate forms films and hydrogels that can absorb up to 20 times their weight in wound exudate, thereby promoting hemostasis and supporting the development of granulation tissue after surgery. Their inherent properties and uniform internal structure also make them effective drug carriers for postoperative repair, further enhancing wound healing [9].

The misuse of antibiotics over six decades has led to widespread antibiotic resistance among pathogenic bacteria. To address this, alternatives to antibiotics are being explored, including natural product compounds, bacteriophage therapy, synthetic compounds, and metal nanoparticles. Among these, natural products—such as plant extracts, essential oils, herbs, and honey—are favored for their low toxicity, accessibility, and consumer acceptance [10,11,12].

Honey is a complex natural substance containing carbohydrates, proteins, amino acids, lipids, vitamins, minerals, and phenolic compounds [13]. It aids wound healing by promoting re-epithelialization and keratinocyte proliferation, supported by its glucose content and essential minerals like zinc and magnesium [14]. With both bacteriostatic and bactericidal properties, honey inhibits microbial growth through dehydration, high sugar concentration, low pH, and hydrogen peroxide production without inducing antibiotic resistance [15]. Incorporating honey into biomaterials enhances its stability and absorption, improving its potential in biomedical applications [16].

Propolis is widely recognized for its antimicrobial, antitumor, anti-inflammatory, and antioxidant properties. Its composition primarily consists of resins and balsams (45–55%), waxes and fatty acids (25–35%), essential oils (10%), and pollen (5%), along with key bioactive components (5%) such as flavonoids, phenolic acids, terpenes, benzophenones, and coumarins, which contribute to its potent biological activity [17]. Propolis exhibits wound-healing and antimicrobial properties, making it a valuable component in biomaterials for skin care and wound dressings. However, its primary limitation is the chemical heterogeneity and complexity, which complicates the standardization of its extracts for consistent use [18].

Royal jelly is a kind of translucent serum with a milky white or light-yellow color and a sweet-sour and astringent taste, secreted from the nutrient glands of the head of worker bees. Its composition is very complex, and the main ingredients are proteins, a variety of amino acids, fatty acids, sugar, vitamins, etc. Royal jelly has anti-inflammatory, antibacterial, antitumor, lowering blood lipids, lowering blood pressure, anti-fatigue, and antioxidant functions [19]. To better understand and harness the potential of natural antibiotics, this study focuses on developing a composite biomaterial that integrates honey, propolis, and royal jelly into a polymeric film.

Therefore, chitosan and alginate were chosen as the polymer matrix due to their exceptional biocompatibility, biodegradability, and intrinsic wound regeneration capabilities. Chitosan’s film-forming ability and its compatibility with bioactive compounds provide a versatile platform for embedding the honey–propolis–royal jelly mixture. The resulting composite material aims to combine the bioactivity of the bee products with chitosan’s structural and functional benefits, creating a synergistic system that enhances wound healing, protects against infections, and promotes tissue regeneration.

This approach seeks to not only overcome the individual limitations of these natural products, such as honey’s low viscosity or propolis’ chemical heterogeneity, but also to offer a scalable, standardized solution for medical applications. This paper focuses on honey and other bee-derived products, such as propolis and royal jelly and especially on their mixture, since in the literature only few investigations are reported on the combined effects of these bee-derived substances. The development of such a polymeric film could revolutionize wound care by providing a multi-functional dressing that supports rapid healing, prevents microbial colonization, and minimizes the reliance on conventional antibiotics, thus addressing the pressing challenge of antimicrobial resistance.

## 2. Materials and Methods

### 2.1. Materials

Sodium alginate, chitosan (low molecular weight), and ethanol (90%) were purchased from Sigma-Aldrich (Darmstadt, Germany). Black locust (*Robinia pseudoacacia* L.) honey and royal jelly were obtained from a personal apiary. Propolis tincture was prepared by dissolving 100 g of propolis from a personal apiary in 1000 mL of 90% ethanol and allowing it to macerate for 48 h.

The antimicrobial assessments were performed using Nutrient Broth No. 2 (NB) and Sabouraud Glucose Agar (Sab), purchased from Sigma-Aldrich (Darmstadt, Germany). All strains tested in this study were obtained from the Microorganisms Collection of the Department of Microbiology, Faculty of Biology, University of Bucharest.

### 2.2. Synthesis of Biomaterials Based on Bee Products

The samples were made from black locust honey, propolis tincture, and pure royal jelly. In order to obtain the mixture, 20 mL of propolis tincture, 5 g of royal jelly, and 35 g of honey were used. The obtained mixture was homogenized in a water bath for 20 min. The mixture was subsequently stored in a dark place in well-sealed containers. For the preparation of sodium alginate chitosan-films, first, the sodium alginate layers were prepared by dissolving honey/propolis tincture/royal jelly or the mixture in 50 mL of distilled water with 1 g of sodium alginate and 0.1 g of glycerol.

**Figure 1:**
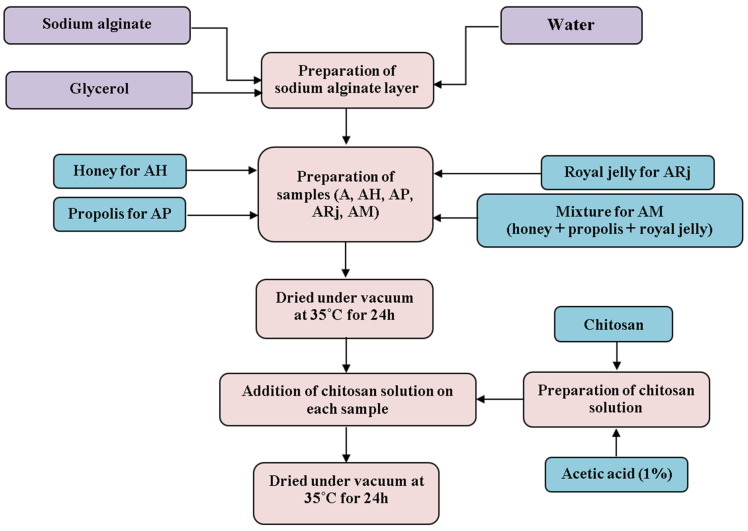
The technological flow of obtaining the samples.

Based on previous literature reports [20,21] and our exploratory experiments we have chosen the following ratios to obtain five samples (Figure 1): A—the control, AH (3.5 g of honey), AP (2 mL of propolis tincture), ARj (0.5 g of royal jelly), AM (5 mL of mixture). The samples were poured into Petri dishes and dried under a vacuum at 35 °C for 24 h. Afterwards, the chitosan solution was prepared by dissolving 2 g of chitosan in 50 mL of 1% acetic acid. 10 mL of chitosan solution was poured onto each layer of sodium alginate and dried under vacuum at 35 °C for 24 h. The composition and label for each sample are presented in Table 1.

### 2.3. Characterization of Biomaterials Based on Bee Products

The surface morphology and microstructure of the films were examined using scanning electron microscopy (SEM) with a QUANTA INSPECT F50 (FEI Company, Eindhoven, The Netherlands).

The spectral data were recorded on the Nicolet iS50 FT-IR spectrophotometer (Thermo Fisher Scientific Inc., Madison, WI, USA) equipped with an ATR device. The scanning range was 4000 cm^−1^ to 400 cm^−1^ at a spectral resolution of 4 cm^−1^ and each spectrum represents the average of 32 scans.

The FTIR 2D maps were recorded with a Nicolet iN10 MX (Nicolet, Waltham, MA, USA) in the domain 4000–650 cm^−1^.

Thermal analysis was made with an STA 449C F3 system, TG-DSC (thermogravimetry-differential scanning calorimetry) from Netzsch (NETZSCH Gerätebau GmbH, Selb, Germany) between 20 and 900 °C in dynamic (50 mL/min) air atmosphere. The evolved gases were transferred through heated transfer line and analyzed on the fly with the help of an FTIR Tensor 27 from Bruker (Bruker Co., Ettlingen, Germany) equipped with an internal thermostatic gas cell.

### 2.4. Antimicrobial Assessments

The qualitatively antimicrobial assays were assessed with the following strains: *Enterococcus faecalis* ATCC 29212, *Staphylococcus aureus* ATCC 25923, *Escherichia coli* ATCC 25922, *Pseudomonas aeruginosa* ATCC 27853, and *Candida albicans* ATCC 10231. The impact of the contaminants on the experiment was mitigated by sterilizing all samples (0.5 cm × 0.5 cm) under UV radiation for 30 min on each side. A sterility test was conducted on each sample by incubating it in NB media for 24 h at 37 °C to confirm the sterility of the films before the antimicrobial assay. The clarity of the broth media confirmed the sterility of the samples. An adapted spot diffusion assay assessed the antimicrobial properties of the newly designed materials [22] according to the Clinical Laboratory Standards Institute [23]. Bacterial and yeast suspensions (1.5 × 10^8^ CFU/mL and 3 × 10^8^ CFU/mL, respectively) were obtained from 24 h cultures on culture media. Petri plates with the specific culture media were seeded with inoculums and each film. The biologically active compounds in the films were used as control solutions (10 µL of each compound). Considering that the tincture and mixture contained 70% ethanol, a control (C_Et_) was performed. The sensitivity of the strains was evaluated after 24 h of incubation at 37 °C, followed by measurement of the growth inhibition zone diameters (GIZD) using ImageJ software (version 1.8.0, National Institutes of Health, Madison, WI, USA).

### 2.5. Biocompatibility Assays

#### 2.5.1. Cell Culture

The human gingival fibroblasts (HFIB-G) cells from Provitro (Berlin, Germany) were maintained in 75 cm^2^ culture flasks using a modified Dulbecco’s Modified Eagle Medium (DMEM) as the growth medium. To enhance cell viability and proliferation, the medium was supplemented with 10% fetal bovine serum (FBS) to provide essential nutrients and growth factors, along with 1% antibiotic–antifungal solution to prevent microbial contamination. The culture medium was replaced every three days to ensure optimal cell growth and metabolic activity. Throughout the experiment, the flasks were incubated in a controlled, humidified environment at 37 °C with a constant CO_2_ concentration of 5%, mimicking physiological conditions to support cell development.

#### 2.5.2. Indirect Cytotoxicity Tests

The samples based on bee products were sterilized using 70% alcohol for 30 min and dried in sterile conditions in the hood. Sample size (h): 1 mm and diameter: 7 mm.

HFIB-G cells were seeded at a density of 2 × 10^4^ cells per well in a 96-well plate, allowed to adhere overnight. After that, the medium was removed and replaced with 200 μL of medium previously incubated for 24 h with the samples based on bee products and then incubated for another 24 h at 37 °C with 5% CO_2_. The control group consisted of cells cultured in DMEM medium without any sample exposure.

Following incubation, the MTT, lactate dehydrogenase (LDH), and nitric oxide (NO) assays were performed to assess cell viability, cytotoxicity, and nitric oxide production.

The MTT assay was used to evaluate cell viability. For this, the 3-(4,5-dimethylthiazol-2-yl)-2,5-diphenyltetrazolium bromide solution (MTT viability assay kit, Biotium, Cat. No. 30006) was utilized. The experiment involved adding 10 μL of MTT reagent to 100 μL of culture medium in each well, followed by a 4-h incubation period. The incubation was followed by solubilization using 200 μL of isopropanol. The absorbance was then measured at 570 nm and 630 nm using the FLUOstar^®^ Omega Multi-Mode Microplate Reader (BMG LABTECH, Ortenberg, Germany) with Omega software version: 5.70 R2.

The LDH cytotoxicity assay was performed to assess cell membrane integrity and cytotoxicity. The assay was conducted using the LDH Cytotoxicity Assay Kit (MAK529 Sigma Aldrich) following the manufacturer’s instructions. For each well, 50 μL of culture medium was mixed with 80 μL of reaction mixture, followed by a 10-min incubation at room temperature in darkness. Absorbance was then measured at 500 nm using the FLUOstar^®^ Omega Multi-Mode Microplate Reader (BMG LABTECH, Ortenberg, Germany).

The NO assay was performed using the Griess Assay to quantify nitric oxide production. The Nitric Oxide Assay Kit, Colorimetric (MAK454) was used according to the manufacturer’s protocol. Working reagent was prepared according to the ratio suggested by the manufacturer (Reagent A: Regent B: Reagent C 100 μL: 4 μL: 100 μL). Samples and working reagent were combined in a ratio of 1:2 and the reaction was run at 60 °C for 10 min. Absorbance measurements were taken at 540 nm using the FLUOstar^®^ Omega Multi-Mode Microplate Reader (BMG LABTECH, Ortenberg, Germany).

### 2.6. Statistical Analysis

The antimicrobial results were statistically analyzed using GraphPad Prism 10.4 from GraphPad Software (San Diego, CA, USA). The data results are expressed as ±SD (standard deviation) and the differences between alginate–chitosan films were analyzed using a one-way analysis of variance (one-way ANOVA), followed by a Tukey’s/Dunnett’s multiple comparisons test. The data were considered statistically significant when the *p*-value was <0.05.

The data results were compared to the values obtained for the control wells and represented graphically. The data used for graph construction represent the arithmetic mean of the results for each type of test. Statistical analysis for biocompatibility assays was performed using Microsoft Office–Excel, applying the standard deviation and *t*-test functions to assess data variability and significance.

## 3. Results and Discussion

### 3.1. Scanning Electron Microscopy (SEM)

**Figure 2:**
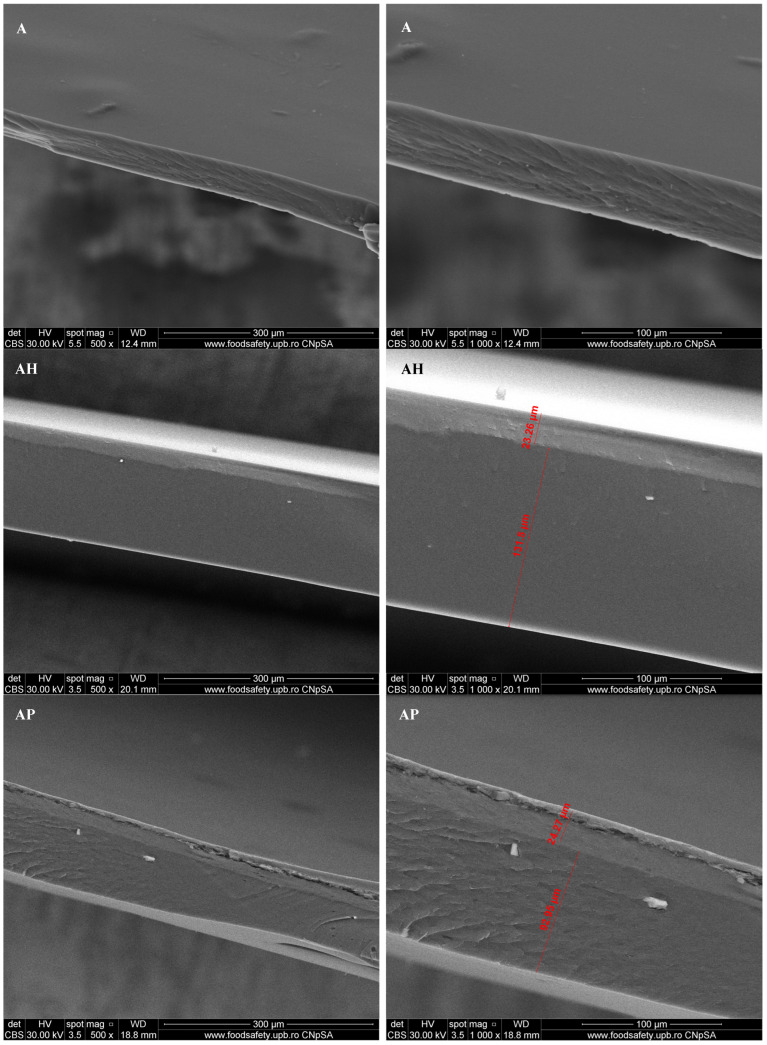

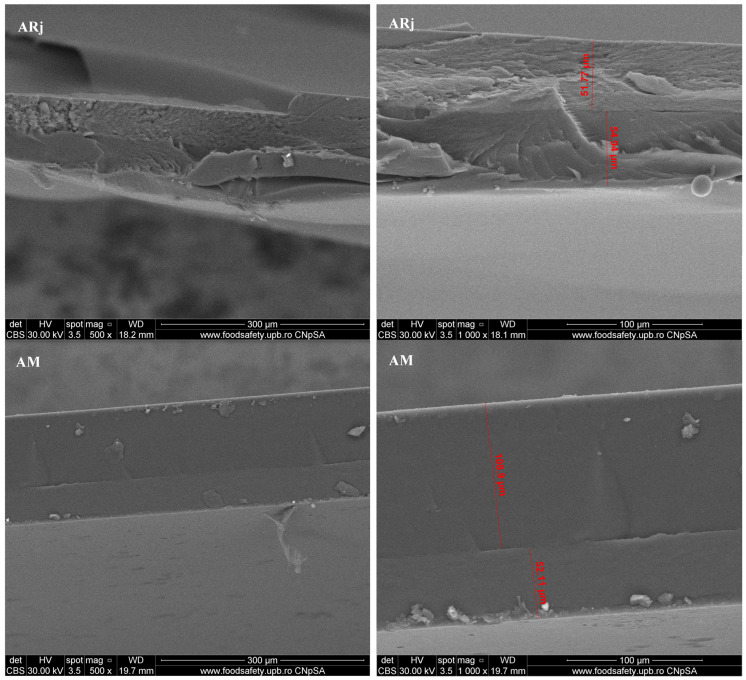
SEM images for composite films A; AH; AP; ARj; and AM.

The SEM analysis of the sodium alginate–chitosan-based composites reveals distinct morphological differences across the various formulations. The control sample (A) exhibits a relatively smooth and compact structure, indicative of a well-formed polymeric blend matrix. The addition of honey (AH) appears to modify the surface slightly, resulting in a smoother texture with some minor porosity, likely due to the incorporation of organic components. Similarly, the sample containing propolis (AP) shows a rougher surface with visible granule-like structures (Figure 2), suggesting phase separation or aggregation of resinous particles [24].

The royal jelly-infused formulation (ARj) demonstrates a fibrous and layered appearance, with areas of increased roughness, likely due to the interaction between the protein-rich royal jelly and the polymer matrix.

In the case of the composite containing a mixture of honey, propolis, and royal jelly (AM), a highly heterogeneous morphology emerges, characterized by different textures and potential phase interactions among the multiple bioactive agents (Figure 2).

Overall, the SEM images highlight the significant influence of bee products on the morphological characteristics of the sodium alginate–chitosan composites, with varying degrees of roughness, porosity, and structural modifications observed across all the formulations.

### 3.2. Fourier Transformed Infrared Spectroscopy (FTIR)

**Figure 3:**
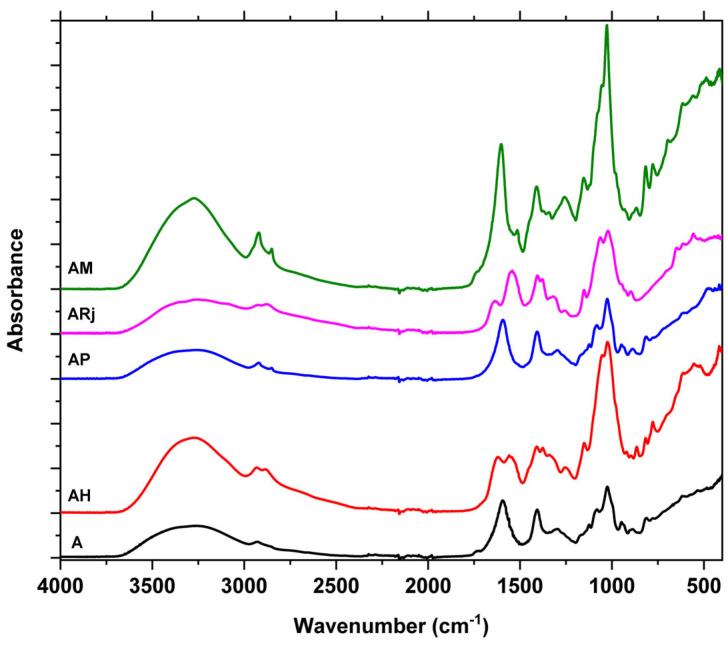
FTIR spectra for the composite films A; AH; AP; ARj; and AM.

The FTIR spectra of the analyzed samples are presented in Figure 3 for easy identification of the modified peaks.

In all five spectra, the broad absorption bands observed at 3260–3280 cm^−1^ are attributed to –OH and –NH stretching vibrations, implicated in intermolecular and intramolecular hydrogen bonding. The double peaks from 2923 cm^−1^ and 2880 cm^−1^ originate from the overlap of –CH symmetric and asymmetric stretching vibrations, which arises due to the presence of –CH_3_ and –CH_2_. As the intensity of the peaks is dependent on the –CH_2_/–CH_3_ ratio, and the polymer matrix contains a higher number of –CH_2_ moieties, the observable peaks are due to –CH_2_ asymmetric (2923 cm^−1^) and –CH_2_ symmetric (2880 cm^−1^) vibrations.

The absorption bands detected at 1594–1603 cm^−1^ correspond to the –OCO– and C=C bonds from the polysaccharides. Additionally, the peaks in this interval can be associated with O–H deformation.

Adding bee products, like honey or royal jelly, leads to the appearance of new peaks at 1619–1638 cm^−1^ due to C=O stretching vibration from glucose and fructose, but also from the amide I band generated by proteins. The peak from 1542 cm^−1^ from AH and ARj (with higher intensity) can be assigned to the amide II band from the proteins. The –CH_3_ asymmetric and symmetric bending deformation are overlapped with the CH_2_ scissor vibration and are visible at 1408 cm^−1^ in all samples. All these vibrational modes are characteristic for carbohydrates, amino acids, and carboxylic acids, which are essential components of honey and royal jelly [25]. These characteristic peaks highlight the molecular interactions and functional groups present in the samples [26].

The 1153 cm^−1^ peak can be assigned to the C–O–C stretching vibration, while the peak from 1023 cm^−1^, visible in all samples, represents C–O stretching (from C–OH moiety). The 1153 cm^−1^ peak, together with the presence of 1254 and 1085 cm^−1^ peaks in AH, ARj, and AM, indicates the presence of an ester.

The peak from 698 cm^−1^ in AH and AM samples indicates the presence of more than four –CH_2_– moieties in a row, but less than ten. It originates from a rocking vibration.

Building on previous research on royal jelly, the characteristic peaks observed in the 1700–1520 cm^−1^ region are associated with protein absorption. Within this range, the amide I band appears between 1700 and 1600 cm^−1^, primarily attributed to C=O stretching vibrations, while the amide II band is detected between 1565 and 1520 cm^−1^, mainly corresponding to N–H stretching vibrations. These spectral features provide valuable insights into the protein composition and structural characteristics of the sample, reinforcing the significance of FTIR analysis in identifying key components [27].

FTIR analysis reveals that propolis is rich in hydrocarbons, phenolic compounds, terpenes, and aromatic hydrocarbons, with lower levels of proteins and carbohydrates. In contrast, royal jelly contains high amounts of carbohydrates and proteins but lower levels of lipids and terpenoids [28].

**Figure 4:**
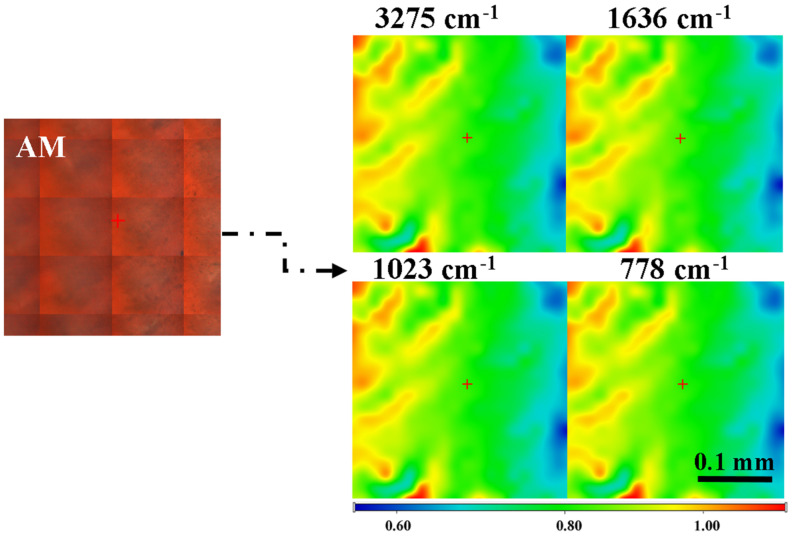
The FTIR maps at 3275 cm^−1^, 1636 cm^−1^, 1023 cm^−1^, and 778 cm^−1^ for the AM sample. Red areas indicate the highest absorbance, while blue areas correspond to the lowest absorbance.

The FTIR microscopy can offer information on the homogeneity of the polymer blend in the films. The chosen wavenumbers are specific to both polysaccharides, alginate and chitosan, but also honey and other organic compounds. Figure 4 presents the FTIR maps for the AM sample at 3277 cm^−1^, 1636 cm^−1^, 1023 cm^−1^, and 778 cm^−1^. The FTIR maps recorded for these specific wavenumbers are similar, indicating that the film is homogeneous, with only minor thickness variation.

### 3.3. Thermal Analysis

**Figure 5:**
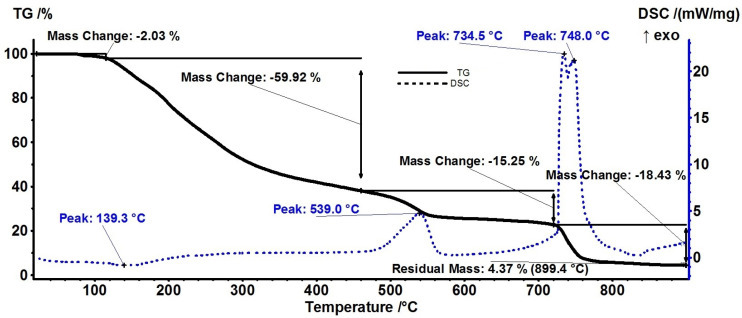
Thermal analysis (TG and DSC curves) for the AM sample.

The thermal analysis was performed on the blended sample (AM) in order to obtain additional information on thermal stability and composition. The AM sample eliminates residual solvent molecules up to 115 °C [29], the recorded mass loss being ~2% (Figure 5).

**Figure 6:**
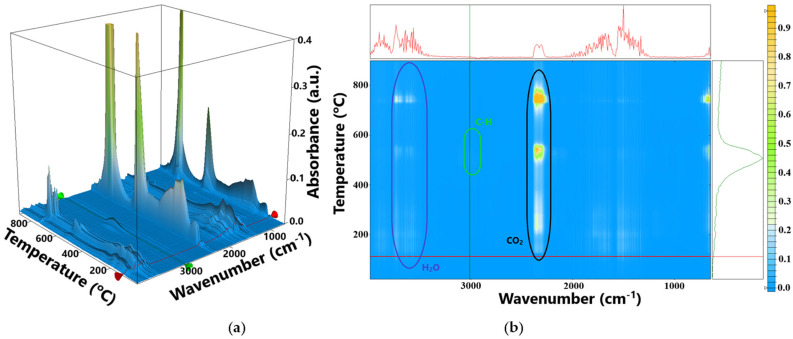
The FTIR 3D diagram for the gases evolved from thermal analysis of the AM sample (**a**); the 2D projections in the temperature/wavenumber plane (**b**); on top of 2D projection is the FTIR spectrum at the temperature of 112 °C, corresponding to the red line in the 3D diagram; at the right side of 2D projection is the evolving trace for the wavenumber 3010 cm^−1^ assigned to the C_sp2_-H vibration from the unsaturated hydrocarbon fragments, corresponding to the green line from the 3D diagram.

The FTIR of the evolved gases (Figure 6) indicates that the carbon dioxide evolving is starting at a low temperature (~115 °C) for the AM sample, most probably by decarboxylation.

The principal degradation process occurs between 115 and 460 °C as a single mass loss step, amounting ~60% of initial mass. The process is a combination of decomposition and oxidation reactions with no net visible effect on the DSC curve beside the weak endothermic effect from 139.3 °C. The evolved gases from this interval indicate the presence of water and carbon dioxide, with some traces of carbon monoxide (Figure 6). The relative intensities of the stretching bands for –OH and C=O indicate that the most probable reactions are the dehydration of the polysaccharides followed by the partial oxidation [30]. The C-H stretching bands are missing from the FTIR spectra up to 460 °C, indicating that no hydrocarbon fragments are eliminated at lower temperatures.

Between 460 and 720 °C, an exothermic effect can be observed on the DSC curve, with a maximum at 539 °C, corresponding to a mass loss of 15.25%. Additionally, in the FTIR 3D diagram (Figure 6) can be observed the evolving of some unsaturated fragments resulted from the breaking up of the polymer backbone, together with larger amounts of carbon dioxide. Therefore, this process can be assigned to the fragmentation of the polymer chains and oxidation of resulting moieties.

After 720 °C, a mass loss of 18.43% is recorded, accompanied by a very strong exothermic peak in the interval 730–750 °C. The FTIR of evolved gases is dominated by the characteristic CO_2_ vibration bands in this temperature interval, indicating the complete oxidation of the residual carbonaceous mass [31]. The principal numeric data are summarized in Table 2.

### 3.4. Antimicrobial Activity

**Figure 7:**
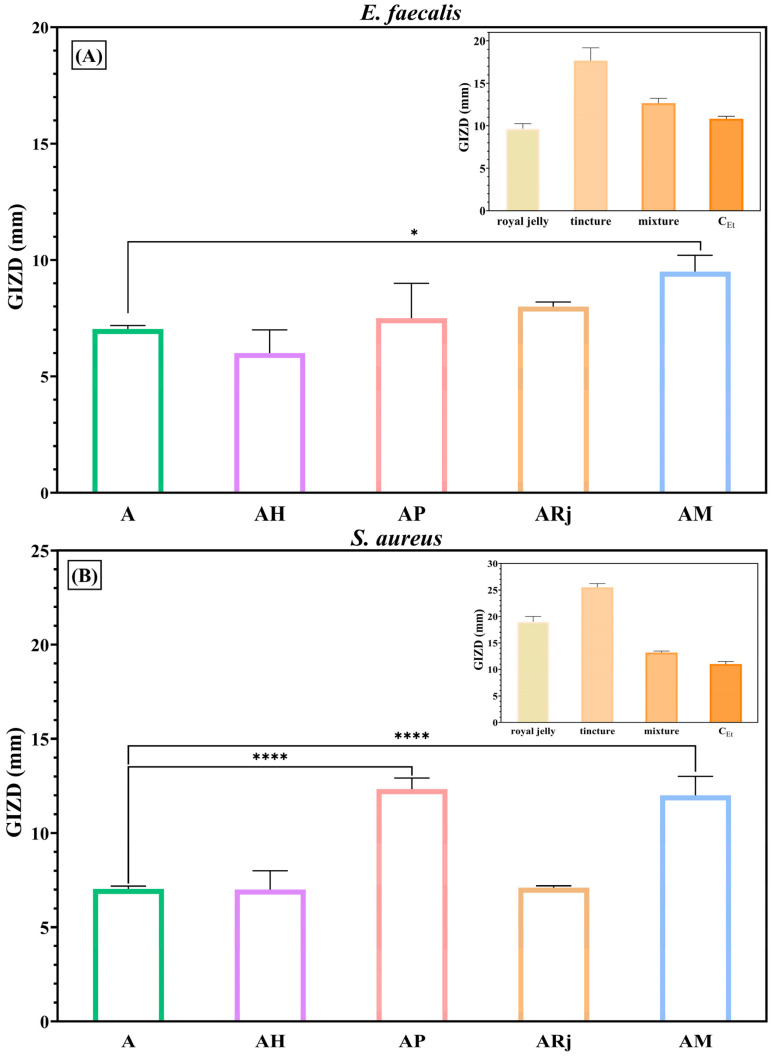
Antibacterial profiles of composite films against Gram-positive bacteria: (**A**) *E. faecalis* ATCC 29212 and (**B**) *S. aureus* ATCC 25923. The data were considered statistically significant (* *p* < 0.05; **** *p* < 0.0001).

**Figure 8:**
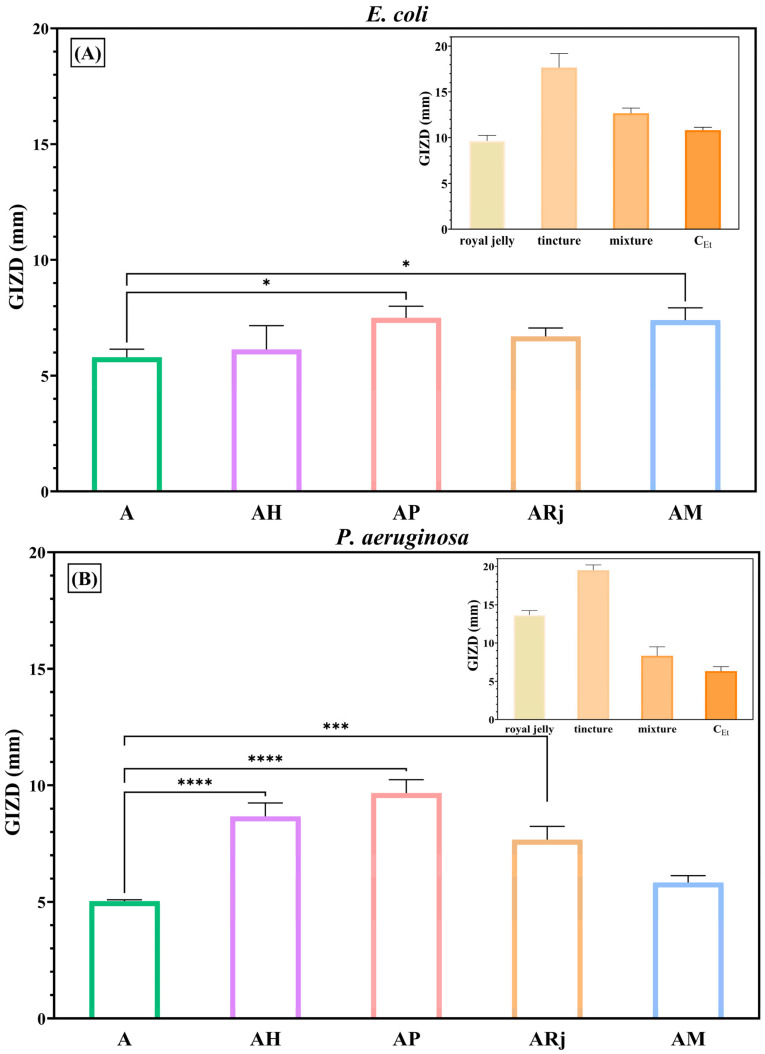
Antibacterial profiles of composite films against Gram-negative bacteria: (**A**) *E. coli* ATCC 25922 and (**B**) *P. aeruginosa* ATCC 27853. The data were considered statistically significant (* *p* < 0.05; *** *p* ≤ 0.0001; **** *p* < 0.0001).

**Figure 9:**
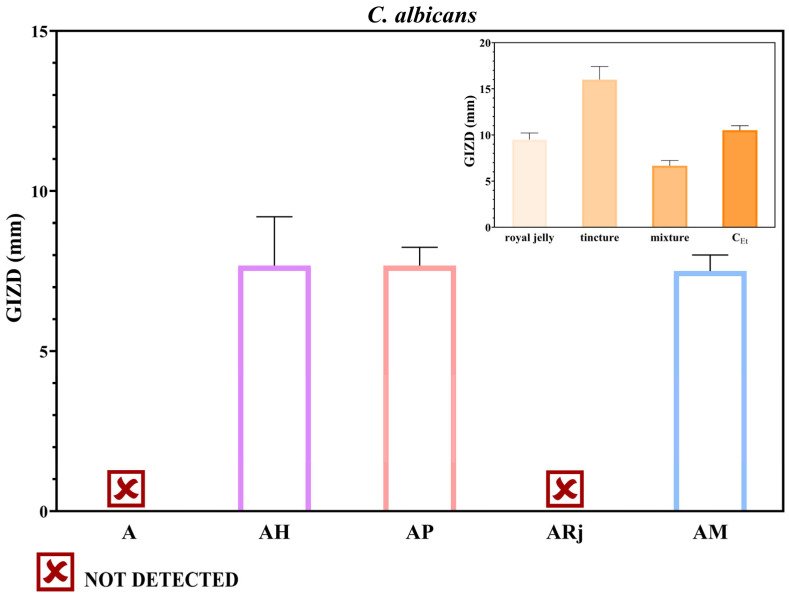
Antifungal profiles of composite films against *Candida albicans*. The data were considered statistically significant (*p* < 0.001).

Firstly, the biological properties of the alginate–chitosan-developed films were evaluated from an antimicrobial point of view. Qualitatively, antimicrobial results were evaluated by measuring the GIZDs that developed near the spot and expressing them as mean values ± SD (Figure 7, Figure 8 and Figure 9).

The alginate–chitosan films displayed a significant inhibitory effect against all tested Gram-positive bacteria. According to Figure 7A,B, the antibacterial patterns for *E. faecalis* and *S. aureus* are similar. Moreover, in the case of *E. faecalis*, the AM sample (alginate–chitosan film with mixture) exhibits a significantly higher inhibition zone than the A sample (pure alginate–chitosan film). In the case of *S. aureus* (Figure 7B), AP and AM samples exhibited the highest activity, with significant differences compared to the A sample (*p* < 0.0001). The antibacterial properties of these samples are attributed to the inhibitory effects of royal jelly and propolis tincture (see inset of Figure 7B). Likewise, Liang et al. [32] showed in their study that royal jelly enhances the antibacterial properties of the chitosan matrix.

The significant impact of alginate–chitosan films against Gram-negative bacteria is shown in Figure 8. Moreover, the samples enriched with propolis tincture (AP) and mixture (AM) presented higher GIZDs than control, A sample. Furthermore, the alginate–chitosan films with honey, propolis tincture, and royal jelly (AH, AP, and ARj) exhibited the highest activity against *P. aeruginosa*, with GIZDs which ranging between 8 and 10 mm. We note that the evaluation of antimicrobial activity was carried out on all beekeeping products, but curiously, honey did not determine zones of inhibition for any of the tested strains. On the other hand, this did not happen in the case of samples with incorporated honey (AH) which exhibited good antibacterial activity, especially against *P. aeruginosa* and *E. coli*. To date, the exact antibacterial mechanism of honey is not fully understood, as it is highly complex and dependent on multiple constituents. In a previous report [33], Deng J.L. et al. found that manuka honey does not inhibit *P. aeruginosa* growth up to a concentration of 70%. In a comparative investigation of black locust, linden, and sunflower honey [34] against *P. aeruginosa*, *H. influenzae*, and *H. parainfluenzae*, the black locust honey was found to have the lowest antibacterial activity among tested varieties. In case of black locust honey, some of the antibacterial activity was attributed to the sugar content in a series of tests against *P. aeruginosa* and *S. pneumoniae* [34]. At the same time, the membrane degradation study indicates that higher water content leads to decrease in the antibacterial activity [34]. In another study made on eight honey types (acacia included), none of them had antibacterial activity against *B. subtilis*, and only three of them manage to inhibit growth of *P. putida* [35].

The literature consensus is that the antimicrobial activity of honey is based on multiple mechanisms [36]. High sugar concentration can inhibit bacteria growth by means of osmotic pressure. At the same time, defensin-1 peptide is partially responsible for the antimicrobial activity. The enzyme glucose-oxidase is involved in the transformation of glucose to gluconic acid and the release of hydrogen peroxide, which is the most potent antibacterial mechanism for honey. However, the enzyme glucose-oxidase is naturally inactive at low pH (the black locust honey used has a pH = 4). Literature reports indicate that the maximum hydrogen peroxide concentration has been observed for honey dilutions between 15 and 50% (*w*/*v*) [37]. Gluconic acid causes a low pH-value in honey, which also helps inhibit bacterial growth. Moreover, some honey types are known for their “non-peroxide” character [38]. For example, the principal antimicrobial compound for manuka honey is considered methylglyoxal [39].

Overall, the antimicrobial efficiency of each honey is dependent on multiple constituents and their concentration, here including the phenolic compounds and the flavonoids. The pH value is an important parameter, as it determines whether these compounds act as antioxidants or have an antibacterial effect, the former behavior being dominant at low pH and the last one manifested at slightly basic pH conditions [40,41].

The lack of zones of inhibition in the case of black locust honey can be explained by the small content of phenolic compounds and by the higher water content, which is eliminated after incorporation into the alginate/chitosan blend. Moreover, previous research has indicated that mixtures of honey and propolis exhibited synergistic antimicrobial effects against various drug-resistant pathogens, indicating that honey’s antimicrobial properties can be amplified through combination with other natural substances [42]. Another investigation showed that medical-grade honey, when supplemented with vitamins C and E, had enhanced antimicrobial activity against *P. aeruginosa* biofilms compared to honey alone, highlighting the potential of honey-based formulations in combating biofilm-associated infections [43]. The observed antibacterial activity of honey after its incorporation into the film matrix suggests that the delivery system plays a crucial role in modulating honey’s bioactivity. Pure honey, when tested alone, may exhibit limited antimicrobial effects due to factors such as dilution during assay preparation, rapid diffusion away from the bacterial cells, or insufficient contact time to exert a significant effect. In contrast, embedding honey within the polymeric film allows for a controlled and sustained release of its active compounds, including methylglyoxal, and various phenolic substances, which are known contributors to honey’s antimicrobial properties [33]. The film matrix can also provide a localized microenvironment that protects these bioactive agents from rapid degradation or dilution, enhancing their stability and prolonging their antimicrobial action. Furthermore, the physical structure of the film may facilitate closer contact between the honey components and bacterial cells, improving adhesion and interaction, which are critical for antimicrobial efficacy. This synergistic effect of honey and the film matrix aligns with previous reports where delivery systems such as hydrogels or nanoparticles have been shown to enhance the antimicrobial activity of natural products by improving their bioavailability and retention at the site of action [44]. Thus, the enhanced antibacterial activity observed in the honey-containing films likely results from a combination of sustained release, protection of active compounds, and improved contact with microorganisms.

Therefore, we confirm that honey’s antimicrobial efficacy can be significantly enhanced when used in combination with other agents or delivery systems, such as alginate and chitosan films, which may facilitate better interaction with microbial communities or improve the sensitization to the active compounds.

The composite films exhibited moderate activity against *C. albicans* (Figure 9). The samples A and ARj did not show the appearance of inhibitory zones. However, the films enriched with honey, propolis, or a mixture presented GIZDs between 7 and 8 mm.

Overall, the composite films exhibited moderate antimicrobial inhibitory effects, and the samples with propolis tincture and the mixture of bee products displayed the highest activity against the tested strains. The potential for these samples in wound treatment should be closely linked to their biocompatibility assessments.

### 3.5. Biocompatibility and Cytotoxicity Assays

#### 3.5.1. MTT Assay

According to Tanaka et al. [45], oral mucosal wound healing follows the same fundamental processes as dermal wound healing, involving cell migration, particularly of oral keratinocytes and fibroblasts during the early phase, but also wound contraction in the later phase, driven by myofibroblasts derived from resident or migratory fibroblasts. In both dermal and oral injuries, fibroblasts play a central role in the deposition and remodeling of the provisional extracellular matrix (ECM). Fibroblasts under the action of TGF-B produce ECM contributing to the restoration of tissue architecture. The expression of collagen and other ECM components is critical for preserving tissue structure and function and is essential for epithelial migration during wound healing and tissue repair. Moreover, oral wound healing is often regarded as an ideal model due to its rapid resolution and lack of scarring.

**Figure 10:**
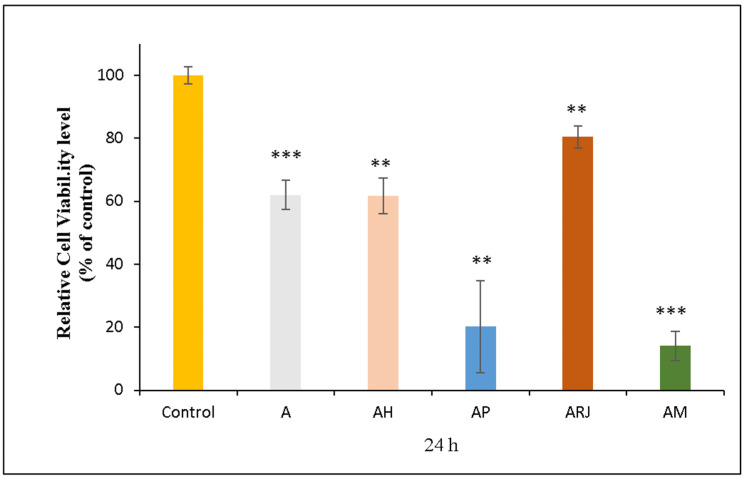
The relative cell viability of human gingival fibroblasts (HFIB-G cell line) measured by MTT assay after 24 h of incubation with medium previously incubated for 24 h with the composite films samples: A, AH, AP, ARj, and AM. Results are expressed as means ± standard deviation (SD) (n = 3) and represented relative to the control (untreated cells). ** *p* < 0.01, and *** *p* < 0.001 compared to control.

The MTT assay evaluates cell viability by measuring metabolic activity, where higher values indicate increased cell survival and proliferation, while lower values suggest cytotoxicity or reduced cell metabolism. The graph from Figure 10 presents the cell viability (% of control) for different composite films, incorporating honey (AH), propolis (AP), and royal jelly (ARj).

The control (~100%) represents the highest cell viability, serving as the control reference. Meanwhile, the A sample (~60%) exhibits a reduction in metabolic activity, indicating a mild baseline cytotoxic effect in the untreated condition.

The AH sample maintains moderate cell viability (~65%), suggesting that honey’s properties contribute to some level of cell survival, despite exerting a mild cytotoxic effect.

The AP sample exhibits a drastic reduction in cell viability, indicating high cytotoxicity. This may be due to propolis’ polyphenols and flavonoids, which, at high concentrations, increase oxidative stress and disrupt cell metabolism. While propolis tincture typically contains a high concentration of ethanol (~70%), the residual ethanol that could be embedded into the AP film could theoretically influence the results. At the same time the drying process was designed to allow for complete ethanol evaporation, and the thermal analysis has not revealed the presence of ethanol among the gases eliminated up to 115 °C, the mass loss of 2.03% being formed from residual water molecules only.

The sample that shows the highest cell viability (~80%) among bioactive films is ARj, suggesting that royal jelly’s peptides, proteins, and fatty acids promote cell survival and metabolic activity, making it a biocompatible formulation.

The AM formulation induces a significant reduction in cell viability, having the lowest viability (~10%), indicating that AM has the lowest biocompatibility, probably because of the effect of propolis, which in our study, in the AP sample, exhibits a low viability (~20%). Propolis sample showed a similar cytotoxicity effect to the human gingival fibroblast (HGF-1) cell line in previous research [46].

The ARj sample has the highest cell viability (~80%), making it a promising candidate for regenerative applications, according to the biocompatibility standard [47].

Our results regarding ARj are in accordance with the data from Yan Lin et al., which demonstrate a good biocompatibility on a human epidermal keratinocyte cell line through the properties to induce a proliferative and migratory effect [48].

Overall, the results suggest that the royal jelly-based sample (ARj) has the highest biocompatibility, while propolis-based film (AP) requires optimization to balance their strong antimicrobial properties with the cytotoxicity.

#### 3.5.2. NO Assay

**Figure 11:**
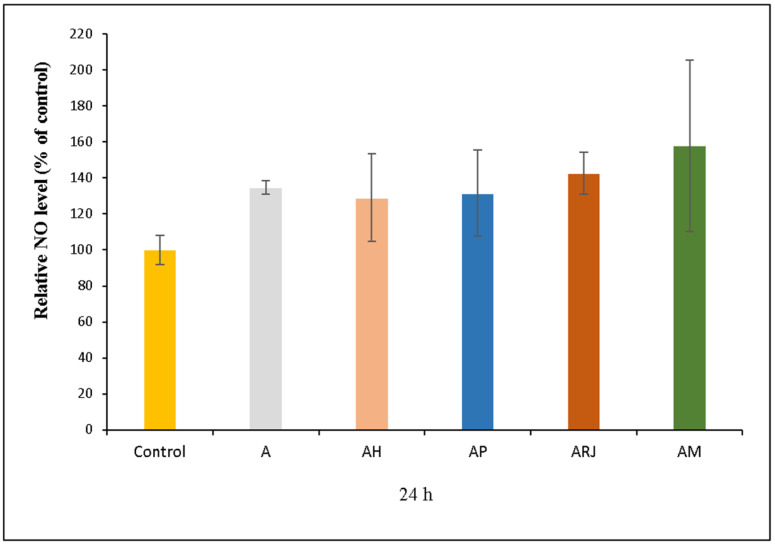
Relative NO level of human gingival fibroblasts (HFIB-G cell line) cells measured by NO assay after 24 h of incubation with medium previously incubated for 24 h with the samples based on bee products: A, AH, AP, ARj, and AM. Results are expressed as means ± standard deviation (SD) (n = 3) and represented relative to the control (untreated cells).

The results of the nitric oxide (NO) assay (Figure 11) provide valuable insights into the immunomodulatory and potential antimicrobial properties of various composite films incorporating bioactive compounds such as honey (AH), propolis (AP), and royal jelly (ARj). Nitric oxide plays a crucial role in immune response, inflammation, and wound healing [49]. The A sample value (200% report to control) indicates a slight immune-modulatory effect of the base material. Among the bioactive film formulations, AH (130%) and AP (130%) induced moderate NO production, likely due to the flavonoids and phenolic compounds present in honey and propolis, which stimulate macrophages. ARj (140%) exhibited a slightly stronger effect, suggesting that royal jelly has a more pronounced immunostimulatory potential compared to honey and propolis.

AM (140%) showed similar NO levels, though its specific bioactivity remains less defined. The biological implications of these results suggest potential applications in wound healing and infection control. Moderate NO production (120–140%) in AH, AP, and ARj supports immune activation without excessive inflammation, making these formulations promising for wound healing. While NO is beneficial for fighting infections, excessive levels could lead to cytotoxicity and prolonged inflammation [50]. However, careful regulation of NO levels is necessary to avoid excessive inflammatory effects.

#### 3.5.3. LDH Assay

**Figure 12:**
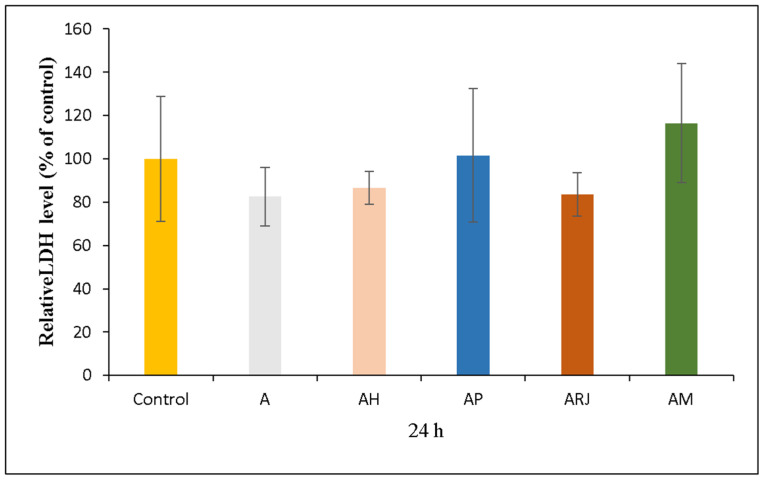
Relative LDH level of human gingival fibroblasts (HFIB-G cell line) cells measured by LDH assay after 24 h of incubation with medium previously incubated for 24 h with the samples based on bee products: A, AH, AP, ARj, and AM. Results are expressed as means ± standard deviation (SD) (n = 6) and represented relative to the control (untreated cells).

In parallel, the LDH assay (Figure 12) provides insights into cell membrane integrity and cytotoxicity. The A sample displayed low LDH release (95% compared to control), confirming that the base material itself is not cytotoxic. Among the bioactive films, AH (85%) and ARj (95%) showed slightly reduced LDH levels, suggesting good biocompatibility with minimal cell membrane damage. AP (100%) maintained an LDH level comparable to the control, indicating neutral cytotoxicity.

However, AM (125%) exhibited an increase level of LDH, showing a substantial loss of membrane integrity. Moreover, these results are correlated to the cell viability results, indicating higher cytotoxicity compared to the control. More than that, in the case of a mixture of honey, propolis, and royal jelly, similar results regarding cytotoxicity have been obtained previously in the literature [51]. Their results revealed that this mixture induced the highest apoptotic effect on tumor hepatic cells.

Considering that only a few studies have been performed in this field of research, further study is required to evaluate the usefulness of such honey mixtures.

Previous studies have shown that hydrogen peroxide, a component generated by some honeys, becomes cytotoxic to human cells at concentrations above 50 μM [33]. On the other hand, manuka honey has been reported to be cytotoxic to human dermal fibroblasts at concentrations exceeding 5% (*v*/*v*), while effective antimicrobial activity typically requires a much higher concentration [52,53]. Our results suggest that honey incorporated into the alginate matrix maintains antimicrobial activity at lower concentrations, potentially reducing cytotoxic risk. The film may serve as a modulated release system, allowing sustained antimicrobial action without surpassing cytotoxic thresholds at the application site. This comparison underscores the importance of delivery method in modulating the therapeutic index—honey alone may require higher (potentially cytotoxic) concentrations, while alginate film incorporation allows us to retain efficacy while minimizing cell damage.

Overall, the results indicate that most formulations show minimal cytotoxicity, suggesting their potential for biomedical applications such as wound healing and antimicrobial treatments. The AH, AP, and ARj-based formulations exhibit controlled LDH release, making them promising candidates for further development. However, AM formulation, with its higher LDH levels, may require concentration adjustments to mitigate potential cytotoxic effects, as the balance between antimicrobial effectiveness and cytotoxicity is a key consideration in designing wound dressings and biomaterials.

## 4. Conclusions

This study aims to develop and evaluate biomaterials derived from bee products and assess their potential effectiveness in soft tissue engineering. The biomaterials were synthesized by combining sodium alginate and chitosan with three distinct bee-derived substances—honey, propolis, and royal jelly—but also with a mixture of these three compounds.

The SEM micrographs of the composite films revealed that the addition of bee products significantly influenced the surface morphology. Variations in roughness, porosity, and microstructural modifications were observed across different formulations, which can influence the microbial adherence capacity, requiring a separate study.

Antimicrobial assays revealed that among the formulated samples, the films enriched with the propolis tincture or bee products mixture exhibited the highest activity against the pathogenic strains. Honey’s antimicrobial efficacy can be significantly enhanced when used in combination with other agents or delivery systems. Additionally, in vitro cytotoxicity studies demonstrated that the AH and ARj samples exhibited minimal toxic effects on cellular viability, indicating their potential biocompatibility. At the same time, the high cytotoxicity of AP and AM samples requires further optimizations as this has become the limiting factor in biomedical applications. Future investigations will focus on optimizing AM formulation by varying the concentration of its key components to enhance its performance in biomedical applications and decrease the cytotoxicity, following in vivo testing.

## Figures and Tables

**Table 1:** Sample composition and labels.

Sample	Alginate	Honey	Propolis	Royal Jelly	Chitosan	Glycerol
A	1 g	-	-	-	0.4 g	0.1 g
AH	1 g	3.5 g	-	-	0.4 g	0.1 g
AP	1 g	-	2 mL	-	0.4 g	0.1 g
ARj	1 g	-	-	0.5 g	0.4 g	0.1 g
AM	1 g	3.5 g	2 mL	0.5 g	0.4 g	0.1 g

**Table 2:** Principal data from the thermal analysis of both samples.

Sample	T1% *	T5% *	T10% *	Mass Loss RT-115 °C	Mass Loss 115–460 °C	Mass Loss 460–720 °C
AM	95.3 °C	132.4 °C	152.7 °C	2.03%	59.92%	15.25%
A	50.1 °C	94.6 °C	156.7 °C	6.94%	53.18%	21.65%

* where Tx% represents the temperature at which the sample has lost 1, 5, or 10%

## Data Availability

The original contributions presented in this study are included in the article. Further inquiries can be directed to the corresponding authors.
